# Cytotoxicity evaluation of carbon-encapsulated iron nanoparticles in melanoma cells and dermal fibroblasts

**DOI:** 10.1007/s11051-013-1835-7

**Published:** 2013-07-24

**Authors:** Ireneusz P. Grudzinski, Michal Bystrzejewski, Monika A. Cywinska, Anita Kosmider, Magdalena Poplawska, Andrzej Cieszanowski, Agnieszka Ostrowska

**Affiliations:** 1 Department of Toxicology, Faculty of Pharmacy, Medical University of Warsaw, ul. S. Banacha 1, 02-097 Warsaw, Poland; 2 Department of Physical Chemistry, Faculty of Chemistry, Warsaw University, ul. L. Pasteura 1, 02-093 Warsaw, Poland; 3 Department of Organic Chemistry, Faculty of Chemistry, Warsaw University of Technology, ul. S. Noakowskiego 3, 00-664 Warsaw, Poland; 4 Department of Clinical Radiology, Faculty of Medicine, Medical University of Warsaw, ul. S. Banacha 1a, 02-097 Warsaw, Poland; 5 Analytic Centre, University of Life Sciences SGGW, ul. J. Ciszewskiego 8, 02-786 Warsaw, Poland

**Keywords:** Carbon-encapsulated iron nanoparticles (CEINs), Cytotoxicity, Melanoma cells, Dermal fibroblasts

## Abstract

Carbon-encapsulated iron nanoparticles (CEINs) are emerging as promising biomedical tools due to their unique physicochemical properties. In this study, the cytotoxic effect of CEINs (the mean diameter distribution ranges 46–56 nm) has been explored by MTT, LDH leakage, Calcein-AM/propidium iodide (PI) and Annexin V-FITC/PI assays in human melanoma (HTB-140), mouse melanoma (B16-F10) cells, and human dermal fibroblasts (HDFs). The results demonstrated that CEINs produce mitochondrial and cell membrane cytotoxicities in a dose (0.0001–100 μg/ml)-dependent manner. Moreover, the studies elucidated some differences in cytotoxic effects between CEINs used as raw and purified materials composing of the carbon surface with acidic groups. Experiments showed that HTB-140 cells are more sensitive to prone early apoptotic events due to raw CEINs as compared to B16-F10 or HDF cells, respectively. Taken together, these results suggest that the amount of CEINs administered to cells and the composition of CEINs containing different amounts of iron as well as the carbon surface modification type is critical determinant of cytotoxic responses in both normal and cancer (melanoma) cells.

**Electronic supplementary material:**

The online version of this article (doi:10.1007/s11051-013-1835-7) contains supplementary material, which is available to authorized users.

## Introduction

Interest in using magnetic nanoparticles (MNPs) has gained a great deal of attention in the last few years following numerous preclinical studies showing that multifunctional MNPs capable of being imaged using magnetic resonance, and loaded with anticancer drugs have offered attractive possibilities for the future improvement of anticancer therapy in humans (Shubayev et al. 2009; Zhang et al. 2009). One major characteristic of MNPs that has made them a really favorite among cancer researchers and biomaterial nanoscientists in general is the ability to modify their surface with different polymers, ligands, and site-specific navigated biomolecules (Ahmed et al. 2012; Davis et al. 2010; Yu et al. 2012; Yuan et al. 2012). In this way, a simple nanoparticle becomes a “smart” nanoparticle to be really used as multifunctional controlled drug delivery systems (DDS) for the personalized cancer nanomedicine (Davis et al. 2010; Yu et al. 2012). Therefore, the innovative approaches to controlled DDS using nanotechnology are revolutionizing the future of cancer treatments in clinical nanomedicine (Hosseinkhani 2006; Subramani et al. 2009; Yu et al. 2012).

In recent years, a number of magnetic nanosystems composing of multifunctional nanoparticles harboring various functions including targeting, imaging, stem cell labeling and tracking have been intensively studied aiming to overcome some limitations associated with conventional cancer diagnosis and therapy (Berman et al. 2011; Hosseinkhani et al. 2012; Miele et al. 2012). This strategy was addressed the “find, fight, and follow” concept of early diagnosis, therapy, and therapy control, sometimes also known as “theranostics” (Xie et al. 2010; Liu et al. 2010; Pan et al. 2009). Among different MNPs studied to date, carbon-encapsulated iron nanoparticles (CEINs) have recently attracted growing interest as a novel generation of magnetic nanomaterial in biomedical and chemical research (Bystrzejewski et al. 2007, 2011; Taylor et al. 2010; Xu et al. 2011; Zeng et al. 2012). These heterostructural nanoparticles are composed of the iron core (generally of the spherical shape), which is covered by carbon coating. The carbon coating is formulated of highly crystalline and curved graphene layers, which protects the core against any oxidization processes, preserves its specific magnetic properties, and further endows the encapsulated iron nanoparticles with the bio-compatibility and stability in many organic and inorganic media including animal and human fluids (Bystrzejewski et al. 2007, 2011). It should be emphasized that carbon-encapsulated MNPs have been also found to be readily susceptible to chemical and biological functionalization (Grass et al. 2007; Park et al. 2011; Poplawska et al. 2010). This feature has opened exciting avenues for using CEINs as novel “smart” contrast agents in molecular magnetic resonance imaging (mMRI) and targeted drug delivery (nano)systems in preclinical cancer studies (Park et al. 2011; Taylor et al. 2010; Tietze et al. 2012; Yoo et al. 2011).

Along with the expanding interest in MNPs in general their potential toxicity has become one of the major concerns. To date, most research programs in the potent toxicity evaluation of iron nanoparticles have focused mainly on iron oxides, such as magnetite (Fe_3_O_4_) and maghemite (γ-Fe_2_O_3_). A number of studies evidences that specific properties of iron oxide nanoparticles such as enhanced reactive area, ability to cross cell and tissue barriers, resistance to biodegradation, amplify their cytotoxic potential relative to molecular or bulk counterparts (Gupta and Gupta 2005; Safi et al. 2011; Tomitaka et al. 2009; Ying and Hwang 2010). Iron oxide nanoparticles have been found to induce oxidative stress, which manifests in activation of reactive oxygen species, followed by a pro-inflammatory response and DNA damage leading to cellular apoptosis and mutagenesis (Naqvi et al. 2010; Novotna et al. 2012; Zhu et al. 2010). Several pharmacokinetic reports indicate that the liver of laboratory animals is the most important organ involving the bioaccumulation and clearance procedures of the iron oxide nanoparticles studied in preclinical models (Schlachter et al. 2011). In general, ultra-smaller superparamagnetic iron oxide (Fe_3_O_4_) nanoparticles, also called as USPIO (under 50 nm in diameter), were found to circulate for longer than larger USPIO particles (over 50 nm), and can be gradually taken up by the reticuloendothelial system in lymph tissue and bone marrow. Larger USPIO nanoparticles, named as SPIONs, are generally taken up quickly by the reticuloendothelial system in Kupffer cells of the liver, and have limited uptake in lymph and bone tissues (Briley-Saebo et al. 2004; Jain et al. 2008; Kim et al. 2006; Van Beers et al. 2001).

In comparison with iron oxide MNPs, the carbon-encapsulated iron nanoplatform containing the iron core covered by a carbon coating has recently been found to alter complex intracellular signaling pathways, resulting in a cascade of possible toxic effects including apoptosis in human hepatoma cells (BEL-7402) (Kai et al. 2011). Studies evidenced that the increase in apoptosis caused by CEINs was accompanied with the Bax over-expression, mitochondrial membrane potential decrease, and the release of cytochrome C from mitochondria into cytosol (Kai et al. 2011). Recent toxicological observations of different groups used for surface coatings and/or modification types in MNPs suggest that some functional groups such as hydroxyl (–OH), carboxylic (–COOH), amine (–NH_2_), and citrate are especially critical determinants of cellular responses, degrees of cytotoxicity, and potential mechanisms of toxicity (Hong et al. 2011; Kai et al. 2011; Unfried et al. 2007). Surface functional groups (–COOH or –NH_2_) also determined the toxic potential of graphene and carbon nanotubes (Jain et al. 2011; Singh et al. 2011). Moreover, carbon-based nanomaterials, such as graphene oxide sheets, have recently demonstrated to be really cytotoxic to the cells and have shown to induce thrombosis upon systemic administration to the animal (Singh et al. 2011). Interestingly, prothrombotic character of graphene oxide sheets was found to be dependent on surface charge distribution as reduced graphene oxide or amine-modified graphene was significantly less effective in aggregating platelets (Singh et al. 2012). To data, amine-modified single-walled carbon nanotubes were also found to protect neurons from injury in rats (Lee et al. 2011). In addition to surface modifications in MNPs, some unique physicochemical properties of nanoparticles, such as shape and size might also play a critical role in producing cytotoxic effects, and plausibly influences aggregation states during in vitro examinations of MNPs and graphene nanoparticles in cell culture models (Akhavan et al. 2012; Hong et al. 2011). It was noted that iron oxide nanoparticles coated with negatively charged citrate ligands were destabilized in cell culture media and interacted strongly with human lymphoblastoid cells (Safi et al. 2011). Studies also evidence that interactions between iron oxide nanoparticles and living cells depend dramatically on the behavior of the nanoparticles in biological fluids. This observation was found in agreement with other recent findings, showing that iron oxide nanoparticles with negative charges on the surface were readily absorbed in cells via receptor-mediated cell endocytosis (Zhang et al. 2008). To our knowledge, by subtle modifying the surface of the CEINs and/or iron oxide nanoparticles, with different aliphatic or aromatic ligands, one plausibly can interfere with the delicate balance of the cellular homeostasis (Hong et al. 2011; Kai et al. 2011; Magrez et al. 2006; Unfried et al. 2007). Therefore, the side and the type of surface functional groups are crucial factors that plausibly determine the biological safety of CEINs, as these factors are hypothesized to be directly related to cytotoxicity, which are pivotal for in vivo practical applications such as DDS and targeted imaging in translation cancer research.

Cellular interaction of MNPs is one of the big challenges in recent nanotoxicology programs (Kim et al. 2012). Although a variety of studies dealing with the cytotoxicity of MNPs exist in recent literatures, so far, no such studies were concerned with CEINs and their cytotoxic effects on melanoma cells. Melanomas are the most aggressive skin cancers in humans, in part, due to their unique ability to rapidly invade the different tissue and organs, thus making anticancer therapy much more difficult (Evans et al. 2013). Melanoma mortality rates of 1.5 are similar in Central and Eastern Europe and Western Europe, although rates vary with a high of 3.2 in Norway and a low of 0.9 in Greece (Forsea et al. 2012). In general, rising incidence rates of cutaneous melanoma have been observed during the last four decades in white populations worldwide (Erdmann et al. 2013). This increase is reported to have leveled off recently in several Northern and Western European countries, Australia, New Zealand, and in North America.

In order to elucidate the CEIN-induced cytotoxicity and the potent mechanism(s) of such deleterious effects, if any due to surface modifications, non-functionalized and pre-functionalized CEINs composing of the modified carbon surface with surface acidic groups bearing different negative charges on the surface were synthesized in a lab scale. The as-obtained CEINs were tested for comprehensive alternative cytotoxicity assays using human (HTB-140) and murine (B16-F10) melanoma cells. Normal human dermal fibroblasts (HDFs) were served as controls. In the light of recent data, especially cell membranes, mitochondria and nucleus have been considered the major cell compartments relevant for possible nanoparticle-induced toxicity (Unfried et al. 2007). Therefore, a dose–response relationship of the carbon-coated iron nanomaterial was elucidated based on the cell membrane, mitochondrial, and nucleus targeted cytotoxicity endpoints (in vitro). The most important question raised in the present study is also whether there is a minimum set of physical and chemical characterization data for magnetic nanomaterials that is required for their realistic characterization in cytotoxicity studies. In the present study, therefore, a complete evaluation of the size, shape, and the surface characterization of CEINs was examined to support toxicity findings in both melanoma cancer and normal cells.

## Materials and methods

### Synthesis and characterization of carbon-encapsulated iron nanoparticles

#### Synthesis and surface modification

CEINs were synthesized by a carbon arc route. The detailed procedure is described elsewhere (Borysiuk et al. 2008; Bystrzejewski et al. 2005). The synthesis method is based on sublimation of the heterogeneous anode containing iron (Fe) and graphite (C). These materials are transformed to the vapor phase congruently due to the very high temperature in the carbon arc (5,000–6,000 K). Next, the as-formed metal–carbon gas undergoes rapid cooling, which leads to nucleation and solidification of iron nanoparticles encapsulated in carbon cages. In brief, the carbon arc discharge was ignited between the graphite cathode and graphite anode, which was doped with Fe (45 wt%). The discharge was maintained under Ar–H_2_ atmosphere (50:50 vol%) at the total pressure of 60.0 kPa. The as-obtained raw product contained CEINs and non-encapsulated iron particles (Fe@C/Fe). A fraction of the Fe@C/Fe sample was further subjected to a purification process to remove the non-encapsulated Fe particles (Fe). The purification procedure included 24 h refluxing in boiling 3 M HCl with further washing in excess of water and ethanol, and drying in air at 70 °C. The purified CEINs were also chemically oxidized to introduce surface acidic groups which are covalently bound to the carbon coatings. The corresponding product was noted as Fe@C–COOH. In a typical run, 2 g of the Fe@C product was suspended in a mixture containing 80 ml of concentrated H_2_SO_4_ and 26 ml of concentrated HNO_3_. The sample was sonicated in a water bath at 25 °C for 3 h (80 W). Then, the suspension was diluted by 1 l of distilled water and allowed to cool down for 2 h. The suspended particles were recovered on a membrane filter under reduced pressure and washed with excess of water and ethanol. To obtain the Fe@C–CH_2_CH_2_–COOH sample, the organic acyl peroxides of dicarboxylic acids, HOOC(CH_2_)_*n*_C(O)OO(O)C(CH_2_)_*n*_COOH (*n* = 2,3) were used as a precursors of the so called “functional” radicals (Peng et al. 2003). Thermal decomposition of succinic acid peroxide resulted in the generation of 2-carboxyethyl radicals (^•^CH_2_CH_2_–COOH). The radicals thus formed are very reactive and ready to functionalize the purified CEINs. The corresponding product was noted as Fe@C–CH_2_CH_2_–COOH. In brief, the purified (Fe@C) was dispersed in *o*-dichlorobenzene (Sigma-Aldrich) under inert atmosphere, heated to 150 °C and succinic acid acyl peroxide was added three times with 24 h intervals. After the reaction the carbon material was filtered through nylon filter, washed with *o*-dichlorobenzene, methanol, and acetone (all from Sigma-Aldrich). The crude material was then sonicated in concentrated HCl for 0.5 h, filtered and washed with water until the eluate became neutral.

#### Characterization

The morphological details of the products were analyzed by transmission electron (TEM) microscopy. The diameter distribution was obtained by analyzing 100–120 objects on TEM images and then the respective histograms were plotted. The iron content was evaluated by thermogravimetry in an oxygen atmosphere. The point of zero charge was determined by the mass titration method (Noh and Schwarz 1989). The pH of a 20 ml 0.01 M NaCl solution was adjusted to 10 by NaOH and HCl. Then, CEIN samples were added successively in 10–50 mg increments and the resulting pH value was measured after reaching an equilibrate pH value (typically after 10–20 min). The solutions were magnetically stirred and purged with Ar. The point of zero charge was determined from the plateau in the plot of pH versus added mass (Supplementary Fig. S1). The measurements were done using a digital pH-meter (Metrohm) calibrated with standard buffers (purchased from POCH Gliwice). The amounts of the surface acidic groups were determined by Boehm titration (Boehm 1994). The CEIN sample of mass ca. 200 mg (Fe@C–COOH, Fe@C–(CH_2_)_2_–COOH) or 600 mg (Fe@C/Fe, Fe@C) was added to a 50 ml vial containing 25 ml of the following solutions: NaOH, NaHCO_3_, and Na_2_CO_3_ (each solution had the concentration of 0.05 M). The vials were tightly protected by a parafilm and shaked for 24 h. Then, the CEINs were removed by a neodymium magnet and filtered. Aliquots (5 ml) were titrated with 0.05 M HCl with continuous Ar purging. All titrations were performed in triplicate using a Metrohm Titrando automatic titrator. The amount of acidic groups was determined under the assumptions that carboxylic, lactonic, and phenolic groups are neutralized by NaOH, carboxylic and lactonic groups are neutralized by Na_2_CO_3_, and carboxylic groups are neutralized by NaHCO_3_.

The surface charge density (SCD), which expresses the mean charge value over the global surface area, which is available to the surrounding environment, was calculated using the formula as follows:

$$ {\text{SCD}} = \frac{{{\text{TC}} \cdot F}}{\text{SA}}[{\text{C}}/{\text{m}}^{2} ] $$

where TC is the total content of surface functional groups determined from Boehm titration (mmol/g), SA is specific surface area (m^2^/g), and F is Faraday constant (96.5 C/mmol). The specific surface area was evaluated from the nitrogen adsorption isotherms.

#### Preparation of CEIN solutions for cytotoxicity studies

In the present study, a purified water solution of carboxymethyl cellulose (CMC, Sigma-Aldrich) was used as a surfactant for CEINs. The stability of CMC suspensions of CEIN samples was evaluated by turbidimetry. The as-sonicated suspension of CEINs was immediately transferred to a quartz cuvette and the absorbance at 532 nm was monitored for 12 h with a step of 1 min using a spectrophotometer (Shimadzu 2401). The obtained absorbance versus time was then converted to concentration versus time curves. The influence of the CMC concentration on the sedimentation stability of CEIN suspensions was also investigated. Three tests were performed, in which the CMC concentration was 0.01, 0.1, and 1.0 mg/ml. The highest stability was found for the largest CMC concentration. In this case the equilibrium concentration of suspended CEINs reached 79 % of the initial concentration. The equilibrium concentration for suspensions prepared in the 0.1 and 0.01 mg/ml CMC solutions was 71 and 60 %, respectively. In the cytotoxicity assay, however, the final 0.1 mg/ml CMC solution was selected to prepare suspensions of all studied CEIN samples, because its viscosity (0.957 mPa s) is comparable to the viscosity of pure water (0.895 mPa s). The viscosity of 1.0 mg/ml CMC solution exceeds more than four times (3.863 mPa s) the viscosity of water.

### Cell culture

Human melanoma cells (HTB-140) and mouse melanoma cells (B16-F10) were used in experiments. Normal HDF was served as controls. All cell lines were obtained from Institute of Oncology (Gliwice, Poland). Frozen stock vials of the cells were thawed and used throughout. Cells were routinely cultured at 37 °C in a humidified atmosphere with 5 % CO_2_ in 75 cm^2^ flasks containing 10 ml of Dulbecco’s modified Eagle’s medium (DMEM), supplemented with 10 % fetal calf serum (FCS), 2 mM l-glutamine, 50 IU/ml penicillin, and 50 mg/ml streptomycin (all from GIBCO). The medium was changed every 3rd day. For subculture, the cells were washed twice with phosphate-buffered saline (PBS) and incubated with trypsin-ethylenediamine tetra-acetic acid (EDTA) solution (0.25 % trypsin, 1 mM EDTA) for 2 min at 37 °C to detach the cells. Thereafter, the complete media were then added into the flask at room temperature to inhibit the effect of trypsin. The cells were washed twice by centrifugation and resuspended in the complete fresh media for reseeding and growing in new culture flasks or plates. Cells were counted using a hemocytometer.

### Cytotoxicity assays

To study the cellular toxicity of the four type of CEINs, the surface non-modified (Fe@C/Fe and Fe@C) and the surface modified (Fe@C–COOH and Fe@C–CH_2_CH_2_–COOH) samples were suspended in a purified water solution containing carboxymethyl cellulose (0.01 % CMC w/v), which served as a surfactant, and then added to the cell culture media and used throughout in all experiments. Respective media used without nanomaterials were served as controls.

#### MTT reduction assay

Cells were seeded on a 24-well plate (NUNC) at a density of 4 × 10^4^ cells/well in 500 μl of DMEM medium supplemented with 10 % FCS and antibiotics (see above). After 24 h of incubation at 37 °C in 5 % CO_2_ atmosphere, the cells were rinsed with the medium, and suspended with fresh medium. To study the cellular toxicity of CEINs, the surface modified and non-modified nanoparticles were added to the cell culture media at concentrations of 0.0001, 0.001, 0.01, 0.1, 1.0, 5.0, 10.0, 50.0, and 100.0 μg/ml, respectively. In control cultures, the cells were placed in 500 μl of the medium without MNPs and CMC at the same cell density. After 24 h of incubation at 37 °C, the nanoparticle suspension was removed, and the cells were washed twice with PBS, detached using trypsin–EDTA solution, and the MTT solution (0.5 mg/ml MTT in PBS) (Sigma-Aldrich) was added for 2 h. Thereafter, the MTT solution was replaced with acidic isopropanol (0.1 N HCl in absolute isopropanol) (Sigma-Aldrich) to dissolve the resulting formazan crystals. Samples were centrifuged at 15,000 rpm for 10 min. Absorbance values at 570 nm were measured within 1 h with a spectrophotometer (UVmini-1240, Shimadzu) after calibration to zero absorbance using acidic isopropanol. The untreated cells were used as a control. The relative cell viability (%) related to control wells containing cell culture medium without nanoparticles was calculated by the following equation: [*A* (test sample)/*A* (control)] × 100 % (where *A* is absorbance).

#### Lactate dehydrogenase (LDH) leakage assay

The leakage of lactate dehydrogenase (LDH) in cells was determined using a cytotoxicity detection kit LDH (Roche). Initially, cells were seeded at a density of 4 × 10^4^ cells/well in a 24-well plate at 37 °C and 5 % CO_2_ atmosphere in 500 μl media per well (DMEM medium supplemented with 10 % FCS and antibiotics). After 24 h stabilization of the cells, the medium in the wells was replaced with the fresh medium containing CEINs at concentrations of 0.0001, 0.001, 0.01, 0.1, 1.0, 5.0, 10.0, 50.0, and 100.0 μg/ml, respectively (test CEIN samples). In low control cultures, the cells at the same cell density were placed in 500 μl of medium without CEINs and CMC. In high control cultures, the cells at the same cell density were placed in 500 μl of medium without CEINs and CMC with 15 μl/well of Triton X-100 lysis solution (Sigma-Aldrich). Additional cell-free wells containing nanoparticles in the concentration range tested were prepared for subtraction of absorption effects. After 24 h exposure, cell culture medium from each well was carefully removed and centrifuged (4,000 rpm, 7 min). Then, aliquots of supernatants (100 μl) were transferred to fresh wells of 96-well plate and mixed with equal amounts of freshly prepared assay reaction mixture containing 30 μl LDH assay solution supported by the assay kit. The microtiter plate was incubated for 30 min at room temperature in the dark as described by the manufacturer. The absorbance was measured at 490 nm with a microplate reader (BioTek, Synergy 4, Biokom) equipped with Gen5 software (BioTech Instruments, Inc., Biokom). The nanoparticle mediated cytotoxicity expressed as the LDH release (%) was determined by the following equation: [(*A* test sample − *A* low control)/(*A* high control − *A* low control)] × 100 % (where *A* is absorbance).

#### Annexin V and propidium iodide staining (apoptosis)

Annexin-V binding was performed using an Annexin-V-FITC kit (Becton–Dickinson) as described by the manufacturer. To data, cells were seeded at a density of 4 × 10^4^ cells/well in a 24-well plate at 37 °C and 5 % CO_2_ atmosphere in 500 μl media (DMEM medium supplemented with 10 % FCS and antibiotics) per well. After 24 h incubation of the cells, the medium in the wells was replaced with the fresh medium containing CEINs at concentrations of 0.001 and 1.0 μg/ml, respectively. After 24 h exposure, the nanoparticle suspension was removed, and detached cells using trypsin–EDTA solution, were washed twice with cold PBS. Thereafter, the cells were then re-suspended in 1× binding buffer after which 100 μl of solution was transferred to a 5 ml culture tube. Annexin V-FITC 5 μl and propidium iodide (PI) 5 μl were added, and the cells were then incubated for 15 min at room temperature in the dark, after which 400 μL of 1× binding buffer was added to each tube and analyzed in the BD FACS Calibur flow cytometer. Flow cytometry analyses were conducted on 5,000 cells in each case. Data analysis was performed with BD FACStation Software (Becton–Dickinson). Apoptosis was quantitatively confirmed by analyzing the percentage of early apoptotic cells using Annexin-V-FITC/PI double staining.

#### Calcein AM and propidium iodide staining (viability and necrosis)

Calcein acetoxymethyl (Calcein AM) and PI staining was performed using a Calcein AM/PI kit (MoBiTec GmbH, Germany) as described by the manufacturer. To data, cells were seeded at a density of 4 × 10^4^ cells/well in a 24-well plate at 37 °C and 5 % CO_2_ atmosphere in 500 μl media (DMEM medium supplemented with 10 % FCS and antibiotics) per well. After 24 h incubation of the cells, the medium in the wells was replaced with the fresh medium containing CEINs at concentrations of 0.001 and 1.0 μg/ml and incubated for 24 h. After removing the culture medium and a gentle washing with PBS, the cells were stained with assay solution containing Calcein AM and PI (2 μl/ml Calcein-AM and 1 μl/ml PI) and incubated at 37 °C for 15 min. Digital images of viable (green fluorescence—excitation wavelength: 490 nm, emission wavelength: 515 nm) and dead (red fluorescence—excitation wavelength: 535 nm, emission wavelength: 617 nm) cells in selected areas were visualized using phase-contrast inverted microscopy (Nikon Eclipse TS 100F) equipped with Nikon Digital Sight DS-U2 camera.

#### TEM analysis (internalization studies)

Cells were seeded at a density of 4 × 10^4^ cells/well in a 24-well plate at 37 °C and 5 % CO_2_ atmosphere in 500 μl media (DMEM medium supplemented with 10 % FCS and antibiotics) per well and allowed to attach for 24 h. After 24 h incubation of the cells, the medium in the wells was replaced with the fresh medium containing CEINs at concentrations of 10 μg/ml and incubated for 24 h. At the end of the exposure, the cells were washed twice with cold PBS and then they were collected and fixed with 2.5 % glutaraldehyde (Serva) buffered in 0.1 M PBS overnight at 4 °C. The samples were washed again with PBS, and post fixed in 1 % osmium tetroxide (Serva) at 4 °C for 1 h. After dehydration in series concentrations of ethanol and infiltration in acetone, cells were embedded in Epon 812 (Serva), and ultra-thin sections cut with glass knives were stained with uranyl acetate (Serva) and lead citrate (Serva), and viewed under JEM 1220 TEM (JEOL).

### Statistical analysis

The data were expressed as mean ± the standard deviation (SD) of three independent experiments. Wherever appropriate, the data were subjected to statistical analysis by one-way analysis of variance (ANOVA) followed by Dunnett’s method for multiple comparisons. A value of *P* < 0.05 was considered significant.

## Results

**Fig. 1:**
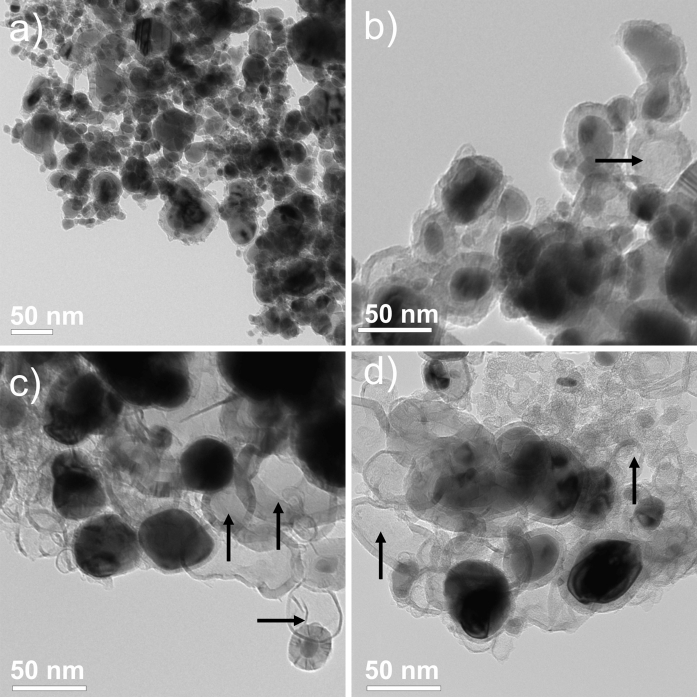
TEM images of Fe@C/Fe (**a**), Fe@C (**b**), Fe@C–COOH (**c**) and Fe@C–(CH_2_)COOH (**d**). Carbon encapsulates with empty core are *arrowed*

**Fig. 2:**
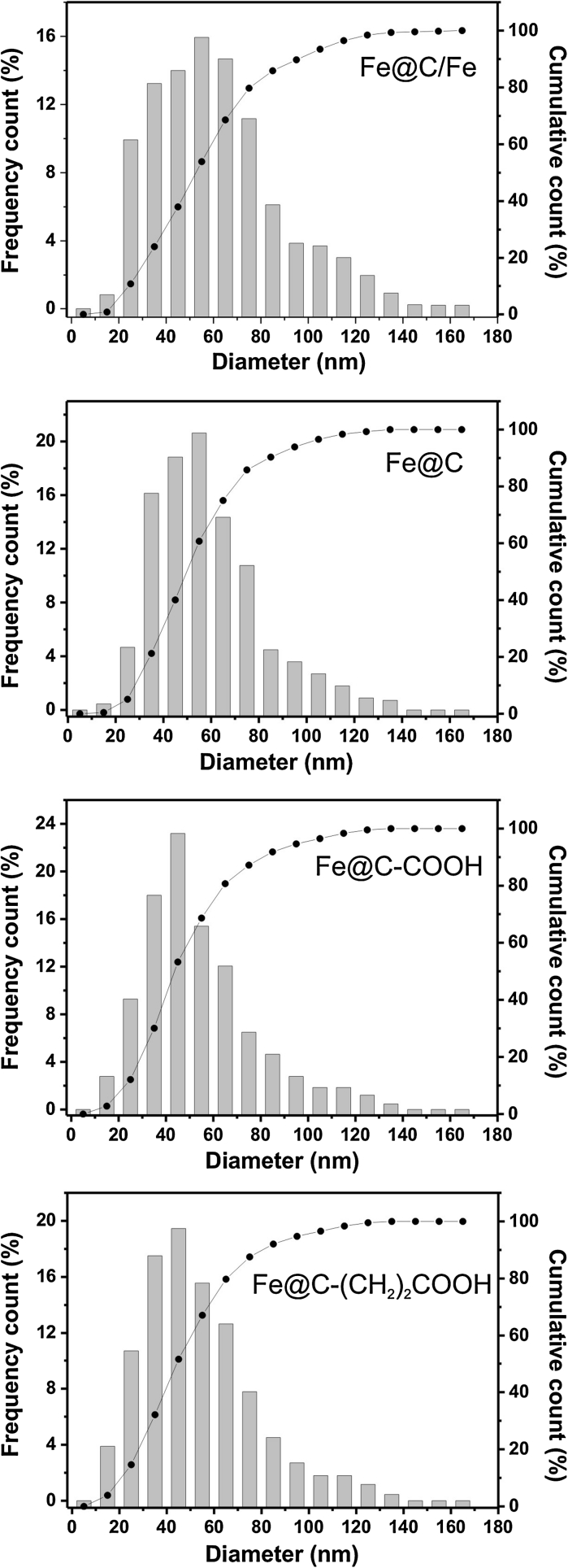
Diameter distribution of carbon-encapsulated iron nanoparticles

The as-obtained raw (Fe@C/Fe), purified (Fe@C), and surface-functionalized CEINs (Fe@C–COOH and Fe@C–CH_2_CH_2_COOH) have similar morphological features. They are composed of spheroidal nanoparticles with a typical core–shell structure, i.e., the metallic cores are tightly covered by a carbon coating (Fig. 1). Their diameter ranges between 5 and 140 nm (Fig. 1). The diameter distributions are presented in Fig. 2. Importantly, the greatest fraction of CEINs has the diameter 40 and 60 nm. The mean diameter values obtained from the histograms are listed in Table 1. This parameter gradually decreases (from 56 to 46 nm) for the purified and surface-functionalized CEINs. This finding indicates that purification and chemical modification result in irreversibly dissolving some of the pristine iron nanoparticles. It is supported in Fig. 1b–d, in which it is shown that some amounts of empty carbon encapsulates are present (examples of these nanostructures are arrowed).

**Table 1:** Weight variation during purification, Fe content, specific surface area, and mean diameter

CEINs	Weight variation (wt%)	Fe content (wt%)	Surface area (m^2^/g)	Mean diameter (nm)
Fe@C/Fe	N.A.	69	60	56
Fe@C	−33	50	77	52
Fe@C–COOH	−47	40	95	46
Fe@C–(CH_2_)_2_COOH	+37	35	75	47

*Fe@C/Fe* the raw CEINs containing carbon-coated iron and uncoated iron particles, *Fe@C* the purified Fe@C/Fe due to removing the uncoated iron particles; *Fe@C*–*COOH* the Fe@C oxidized in H_2_SO_4_/HNO_3_, *Fe@C*–*CH*_*2*_ *CH*_*2*_ *COOH* Fe@C functionalized by attack of 2-carboxyethyl (^.^CH_2_CH_2_COOH) radicals

The chemical treatment (purification or surface modification) of CEINs leads to substantial reduction of iron (Fe) content (Table 1). In the case of Fe@C samples, the observed decrease is to the irreversible leaching of the Fe nanoparticles from the as-obtained product, which are not completely encapsulated in carbon and therefore these particles readily dissolve in an acid. In fact, it is consistent with a TEM image (Fig. 1b) showing some empty carbon encapsulates, which originally had the iron core. On the basis of the data from Table 1, one can state that ca. 28–29 % Fe particles (in relation to the total Fe content) in the raw sample Fe@C/Fe may have a direct contact with the surrounding environment (e.g., components of the biological fluids, cell membranes, etc.). This number is in good agreement with the observed weight loss after purification (33 wt%, Table 1). The CEINs of the sample Fe@C have very high stability in acid media and do not undergo subsequent leaching. The reference test has shown that 10 day storage of the purified CEINs (Fe@C) in concentrated HCl results in 4 % mass loss only (data not shown). The Fe content in samples Fe@C–COOH and Fe@C–CH_2_CH_2_COOH is lower in comparison with purified CEINs. In the case of CEINs functionalized via H_2_SO_4_/HNO_3_ treatment the decreased Fe content is due to the highly oxidizing power of the acids used. The treatment in H_2_SO_4_/HNO_3_ mixture also destroys (to some degree) and perforates the carbon layers in these encapsulate, which have the thinnest coatings. Obviously, the perforation enables the leaching of the iron core and finally leads to the decreased Fe content. This finding is supported by a TEM image (Fig. 1c), which shows empty carbon encapsulates. The sample Fe@C–CH_2_CH_2_COOH contains less Fe in comparison to the purified product (Fe@C). This is an expectable finding, because the covalently attached linkers (37 wt%) contribute to the total mass of the functionalized CEINs (Table 1). Interestingly, the surface functionalization by 2-carboxyethyl radicals is also accompanied by partial perforation of carbon coatings (Fig. 1d).

The results of Boehm titration and values of point of zero charge are presented in Table 2. The samples have substantially different surface chemistry features. The raw CEINs (Fe@C/Fe) have the lowest content of surface groups, which can be neutralized by the strong base (NaOH). This sample has the point of zero charge of 7.4. The observed surface charge in Fe@C/Fe may originate both from (i) partially oxidized non-encapsulated Fe nanoparticles and (ii) oxygen acidic sites on carbon coatings in CEINs. It should be noted that non-encapsulated Fe particles are in continuous contact with the ambient atmosphere and may be oxidized. The measured value of the point of zero charge is significantly lower in comparison to the values reported for pure goethite (FeO(OH); 9.4) (Kosmulski et al. 2003) and zero valent iron nanoparticles (Sun et al. 2006). This comparison supports the above hypothesis concerning that CEINs and non-encapsulated Fe nanoparticles contribute to the net surface charge. The surface of Fe@C, Fe@C–COOH, and Fe@C–(CH_2_)_2_COOH has acidic sites. It is demonstrated by the point of zero charge values, which are lower than 7. Importantly, these samples differ greatly in the total content of surface acidic groups, which changes in the same order as the point of zero charge (Table 2). The smallest acidity (0.16 mmol/g) is found for the purified CEINs (Fe@C). Since this sample has not been subjected to surface functionalization, the acidic sites likely originate from oxygen, which could be bound during the synthesis process (e.g., from contaminations from the buffer gases). The surface modification using the H_2_SO_4_/HNO_3_ mixture increases the total acidity by 7 times. The greatest increase of the surface acidity (almost 16 times) is observed in Fe@C–(CH_2_)COOH sample. The distribution of acidic groups onto a surface of oxidized CEIN samples is slightly different. The carboxylic groups are the most abundant component of the total surface acidity and their relative fraction is 0.47 and 0.56 for Fe@C–COOH and Fe@C–(CH_2_)_2_COOH, respectively. The relative amount of lactonic- and phenolic-like groups in both samples is almost the same.

**Table 2:** Point of zero charge, results of boehm titration, surface charge density, and average density of surface acidic groups (D)

CEINs	Point of zero charge	Total content of functional groups (mmol/g)	Carboxylic groups (mmol/g)	Lactonic groups (mmol/g)	Phenolic groups (mmol/g)	Surface charge density (C/m^2^)	D [groups/hexagon]
Fe@C/Fe	7.42	0.05	<LOD	<LOD	<LOD	0	0
Fe@C	6.71	0.16	0.02	0.06	0.08	0.20	0.21
Fe@C–COOH	4.50	1.11	0.53	0.30	0.28	1.12	1.20
Fe@C–(CH_2_)_2_COOH	4.18	2.51	1.42	0.59	0.50	3.23	3.43

Data are mean (*n* = 3), the observed relative deviation was not greater than 5 %

**Fig. 3:**
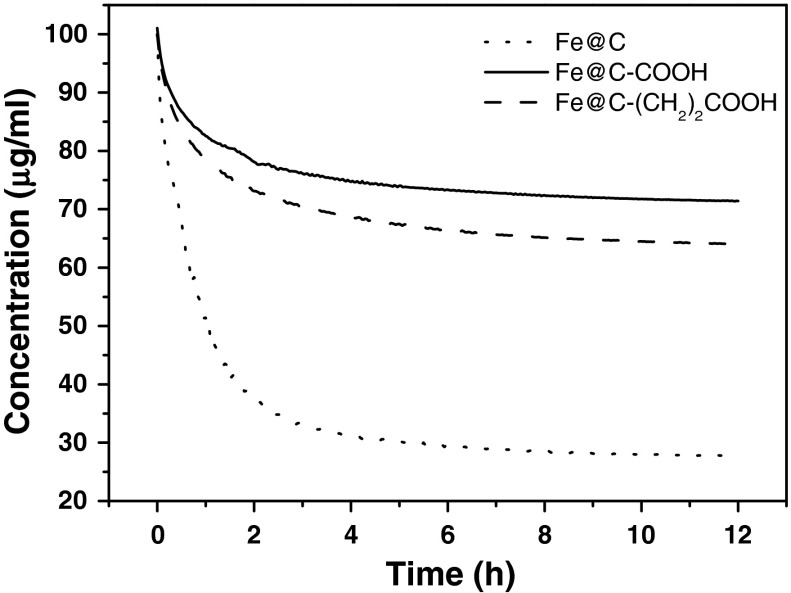
Time sedimentation curves of water CMC (0.1 mg/ml) dispersions of Fe@C, Fe@C–COOH, and Fe@C–(CH_2_)_2_COOH nanoparticles

The stability of CMC (0.1 mg/ml) suspensions of CEIN samples was evaluated by turbidimetry before the cytotoxicity study. Importantly, in the case of sample Fe@C/Fe it was not possible to obtain a stable suspension, even for long (>1 h) sonication times. This drawback results from the highly hydrophobic character of sample Fe@C/Fe (this sample contain uncoated Fe particles, which increase the hydrophobicity). For other CEIN samples, i.e., Fe@C, Fe@C–COOH, and Fe@C–(CH_2_)_2_COOH, respectively, this problem was not observed. The time sedimentation curves are shown in Fig. 3. In each case the concentration gradually decreases with time. The highest drop (75 %) is observed for sample Fe@C. The functionalized CEINs (Fe@C–COOH, and Fe@C–(CH_2_)_2_COOH) have substantially larger stability. The equilibrium concentration is of 70–75 % of the initial concentration. This effect is undoubtedly caused by the presence of surface functional groups, which increase the hydrophilicity and improve the interactions with the surfactant.

**Fig. 4:**
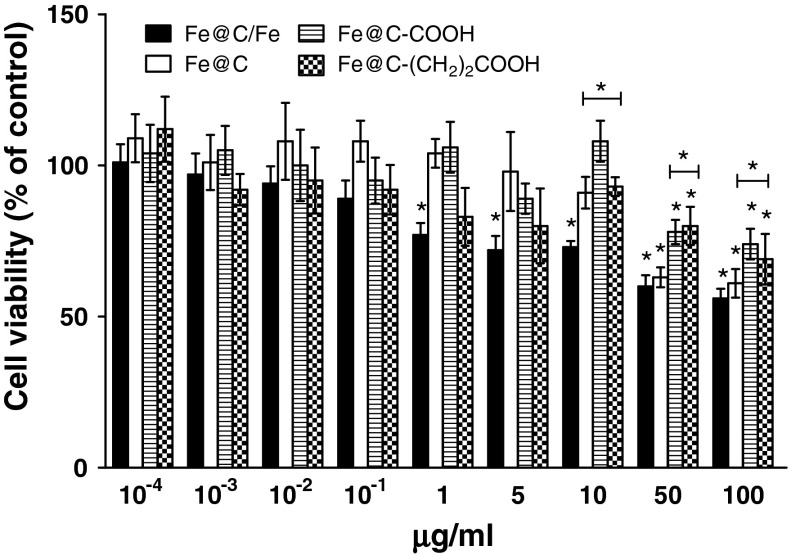
Effect of carbon-encapsulated magnetic nanoparticles on mitochondrial function in human melanoma (HTB-140) cells. Cell viability was determined by MTT reduction assay after 24 h of exposure to CEINs (0.0001–100 μg/ml). Data are mean ± SD. Significant ANOVA test among different CEIN types versus Fe@C/Fe is represented by bracket with a *asterisk* over each data set (*P* < 0.05), *asterisk* above nanomaterial columns indicates statistically significant difference compared to untreated controls (*P* < 0.05). See Table 1 for CEINs labeling

**Fig. 5:**
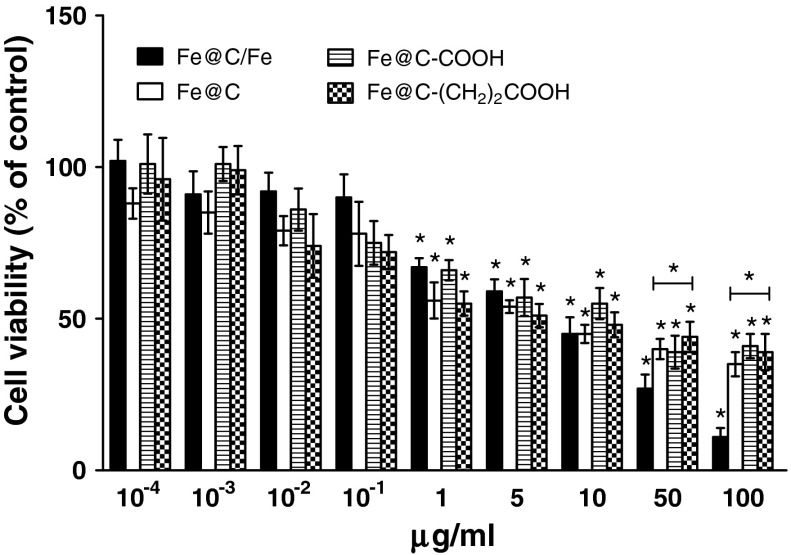
Effect of carbon-encapsulated magnetic nanoparticles on mitochondrial function in murine melanoma (B16-F10) cells. Cell viability was determined by MTT reduction assay after 24 h of exposure to CEINs (0.0001–100 μg/ml). Data are mean ± SD. Significant ANOVA test among different CEIN types versus Fe@C/Fe type is represented by bracket with a *asterisk* over each data set (*P* < 0.05), *asterisk* above nanomaterial columns indicates statistically significant difference compared to untreated controls (*P* < 0.05). See Table 1 for CEINs labeling

**Fig. 6:**
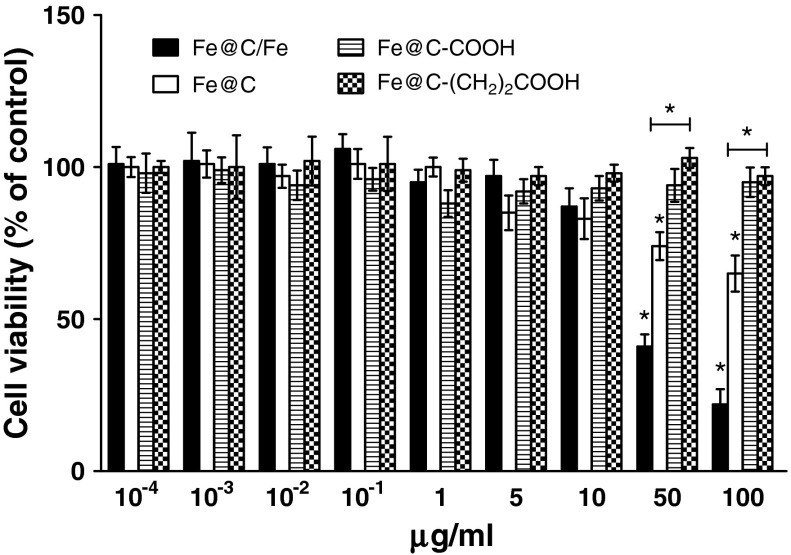
Effect of carbon-encapsulated magnetic nanoparticles on mitochondrial function in human dermal fibroblasts (HDFs). Cell viability was determined by MTT reduction assay after 24 h of exposure to CEINs (0.0001–100 μg/ml). Data are mean ± SD. Significant ANOVA test among different CEIN types versus Fe@C/Fe type is represented by bracket with a *asterisk* over each data set (*P* < 0.05), *asterisk* above nanomaterial columns indicates statistically significant difference compared to untreated controls (*P* < 0.05). See Table 1 for CEINs labeling

The results demonstrate that all four types of CEINs were able to affect the viability of normal and cancer cells in different patterns. After 24 h treated with a raw CEIN material (Fe@C/Fe) at 1–100 μg/ml different concentration, the viability of human and murine melanoma cells decreased as determined by MTT reduction assays (Figs. 4, 5). The studies show that only doses of 50 and 100 μg/ml were enabled to diminish the viability of normal HDFs (Fig. 6). In both human and murine cancer cells, a quite similar profile of the viability was observed when the purified sample Fe@C was introduced at the two large doses (Figs. 4, 5). It should be noted, however, that murine melanoma cells were found to be more sensitive to the sample Fe@C as compared to that of the human ones (Fig. 5). In contrast to normal human fibroblasts, the CEINs functionalized via H_2_SO_4_/HNO_3_ treatment, showed more diminished effects on the cell survival in human and murine melanoma cells (Figs. 4, 5, 6). In general, these patterns were also observed when the carbon surface of the CEINs has been modified by attack of 2-carboxyethyl (^.^CH_2_CH_2_COOH) radicals (Figs. 4, 5).

**Fig. 7:**
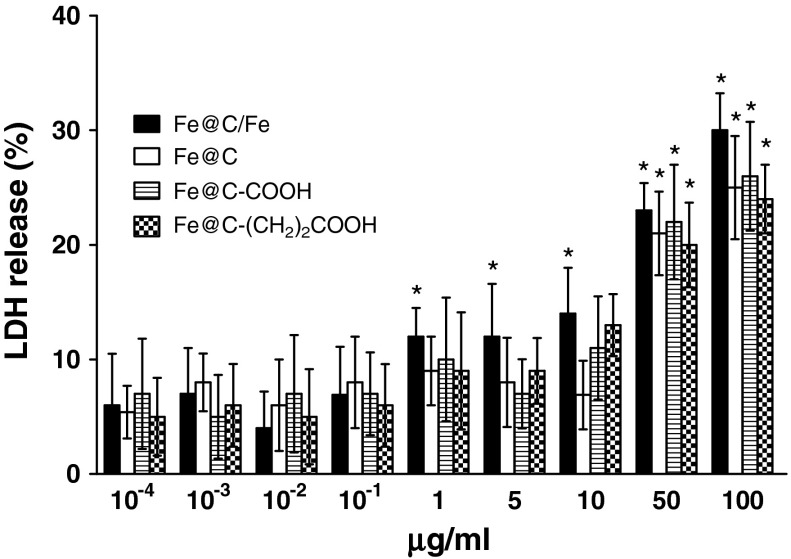
Effect of carbon-encapsulated magnetic nanoparticles on LDH leakage in human melanoma (HTB-140) cells. Cytotoxicity was determined by LDH release after 24 h of exposure to CEINs (0.0001–100 μg/ml). Data are mean ± SD. *Asterisk* above nanomaterial columns indicates statistically significant difference compared to untreated controls (*P* < 0.05). See Table 1 for CEINs labeling

**Fig. 8:**
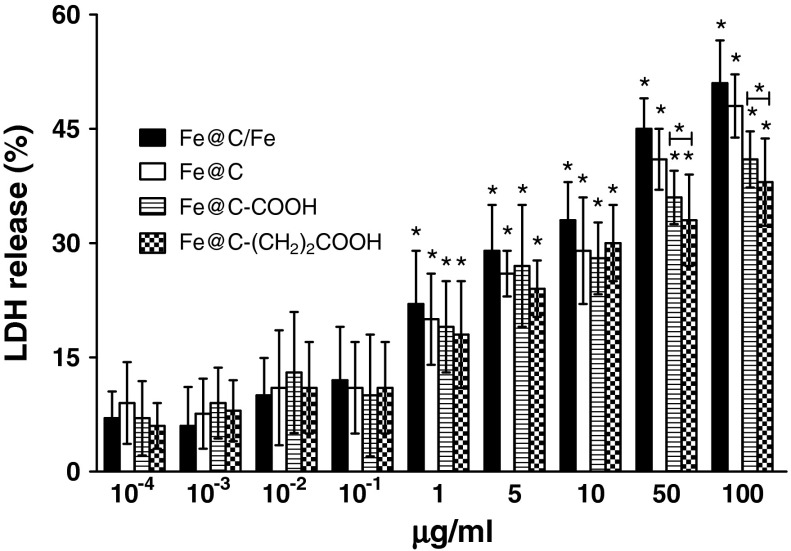
Effect of carbon-encapsulated magnetic nanoparticles on LDH leakage in murine melanoma (B16-F10) cells. Cytotoxicity was determined by LDH release after 24 h of exposure to CEINs (0.0001–100 μg/ml). Data are mean ± SD. Significant ANOVA test among different CEIN types versus Fe@C/Fe type is represented by bracket with a *asterisk* over each data set (*P* < 0.05), *asterisk* above nanomaterial columns indicates statistically significant difference compared to untreated controls (*P* < 0.05). See Table 1 for CEINs labeling

**Fig. 9:**
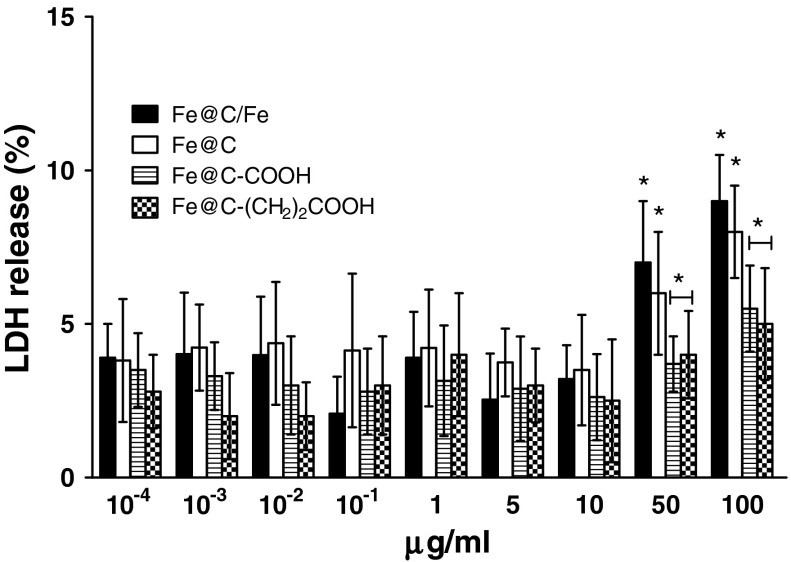
Effect of carbon-encapsulated magnetic nanoparticles on LDH leakage in human dermal fibroblasts (HDFs). Cytotoxicity was determined by LDH release after 24 h of exposure to CEINs (0.0001–100 μg/ml). Data are mean ± SD. Significant ANOVA test among different CEIN types versus Fe@C/Fe type is represented by bracket with a *asterisk* over each data set (*P* < 0.05), *asterisk* above nanomaterial columns indicates statistically significant difference compared to untreated controls (*P* < 0.05). See Table 1 for CEINs labeling

**Fig. 10:**
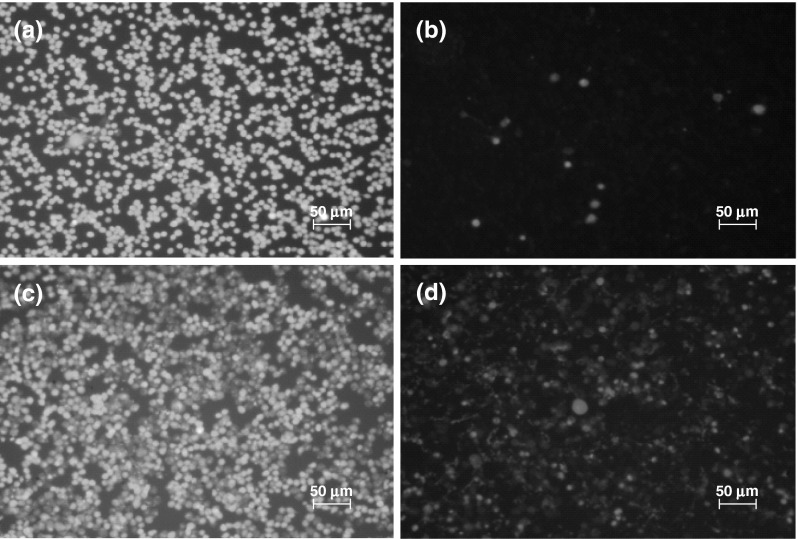
Representative fluorescence images of murine melanoma (B16-F10) cells. Green images (*λ*_ex_ = 490 nm) represent cells stained by acetoxymethyl calcein, and red images (*λ*_ex_ = 545 nm) represent cells stained by propidium iodide. Control untreated cells (**a**, **b**), and Fe@C–COOH treated cells (**c**, **d**) at 1 μg/ml for 24 h. (Color figure online)

After 24 h exposure at varying doses of CEINs, LDH leakages resulted in a different dose-dependent pattern depends on the cell line type and the nanoparticle type and concentrations in medium (Figs. 7, 8, 9). In our results, CEINs with or without surface functional groups elucidated quite similar profiles in LDH leakage assays on human and murine cancer cells (Figs. 7, 8). However, the release of LDH in murine cancer cells exposed to all type of CEINs was higher than that in human cancer cells (Figs. 7, 8). Interestingly, the LDH release profile in HDFs exposed to all fore type of CEINs were around 5 % at the concentration of 0.0001–50 μg/ml, respectively (Fig. 9). When the CEINs concentrations increased up to 100 μg/ml, the increased level of LDH release was observed (Fig. 9). This membrane effect of CEINs was also studied using a Calcein AM/PI double staining method, which mainly describes on cell viability due to membrane permeability assays. In accordance with data from both MTT reduction and LDH leakage assays, the results of our studies also confirm that PI positive staining was slightly increased in human melanoma cells (data not shown), and especially, in murine cancer cells exposed to all four type of CEINs that evidenced that a number of such cells are expected to be damaged or dead in 24 h-post treatment period (Fig. 10). PI is a fluorescent vital dye that stains DNA. It does not cross the plasma membrane of cells that are viable or in the early stages of apoptosis because they maintain plasma membrane integrity. In contrast those cells in the late stages of apoptosis or already dead have lost plasma membrane integrity and are permeable to PI (Fig. 10).

**Fig. 11:**
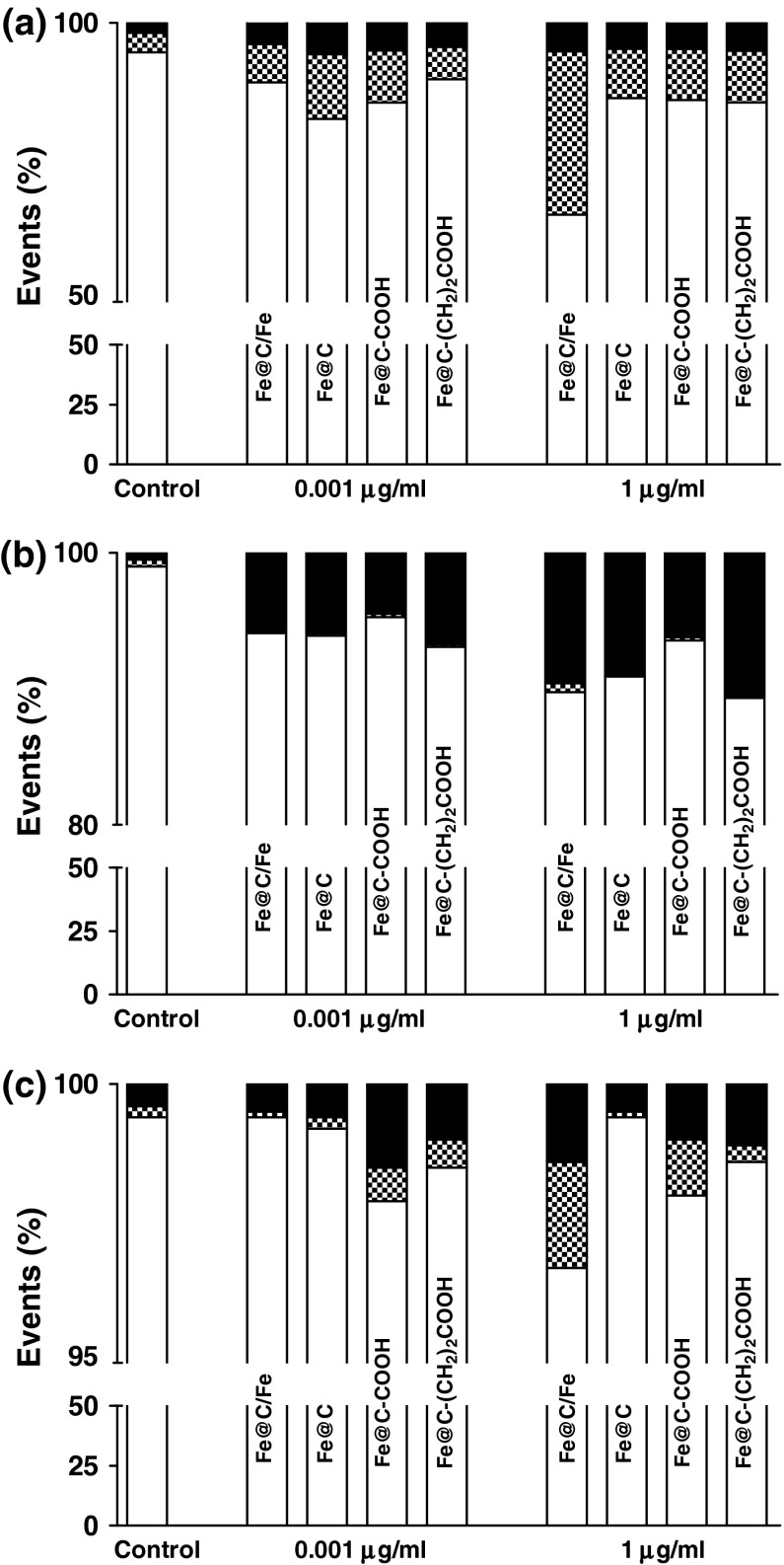
Flow cytometric analysis of human melanoma HTB-140 (**a**), murine melanoma B16-F10 (**b**), and human dermal fibroblasts HDFs (**c**). Cells were exposed to carbon-encapsulated iron nanoparticles for 24 h. Cells were double staining with Annexin V-FITC and propidium iodide, and then fixed and analyzed on a flow cytometer. *White bars* life cells, *dashed bars* early apoptosis, *black bars* late apoptosis or necrosis

To assess the extent and mode of early or late apoptotic events induced by CEINs with or without acidic groups on the carbon surface, Annexin-V-fluorescein isothiocyanate (Annexin-V-FITC) and PI double staining was performed using a FACS method in studies. To data, the externalization of phosphatidylserine (PS), which is localized commonly on the outer cellular membrane, was monitored as a key step in early stage apoptosis. Annexin-V which has a strong calcium-dependent affinity for PS was applied to determine the apoptotic rate of the tested cells in response to the treatment of CEINs. In the present study, the Annexin-V(−)/PI(−) cell population was regard as normal cells, while positive staining just for Annexin-V(+) but negative staining for PI(−) was nominated as a measure of early apoptosis, and Annexin-V(+)/PI(+) was nominated to late apoptosis or necrosis. As shown in Fig. 11a, compared with the untreated control of human melanoma cells, a significant increase in the ratio of early apoptosis cells was observed in both raw CEINs and purified CEINs with or without carboxylic groups, respectively. Murine melanoma cells were found to be more sensitive to prone late apoptosis or necrosis events (Figs. 10, 11b) as compared to that of human melanoma cells (Fig. 11a) and of normal HDFs, respectively (Fig. 11c).

**Fig. 12:**
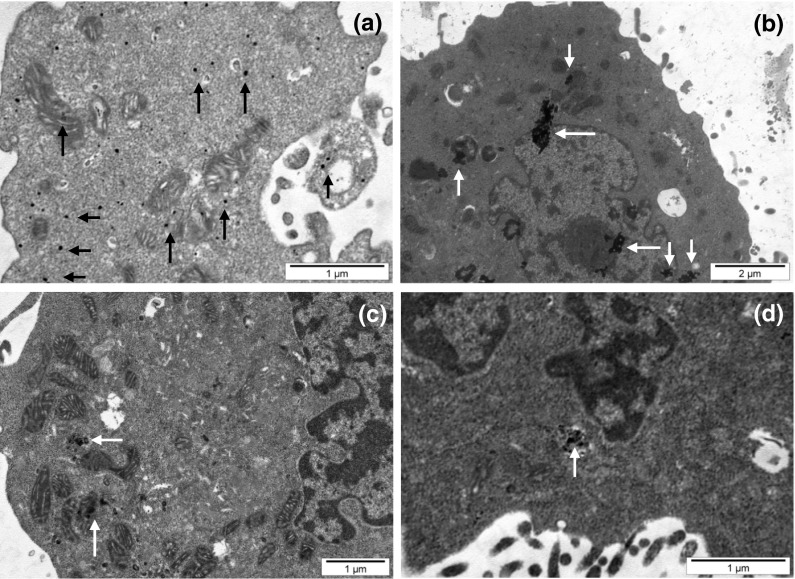
Representative TEM images of murine melanoma (B16-F10) cells exposed to carbon-encapsulated iron nanoparticles at 10 μg/ml for 24 h. *Arrows* show Fe@C (**a**, **b**, **c**) and Fe@C–CH_2_CH_2_COOH (**d**) nanoparticles internalized in different cellular compartments and organelles such as cytoplasma and mitochondria (**a**, **c**) and nucleus (**b**, **d**)

Our TEM studies clearly evidenced that all kinds of CEINs were incorporated into melanoma cells after 24 h incubation at 10 μg/ml. As shown in Fig. 12a, b the purified CEINs (Fe@C) were distributed both as single nanoparticles and conglomerates on the cell membrane and inside of cell penetrating within the organelles such as nucleus and mitochondria. In B16-F10 cells showed a large body of chromatin condensation, typical of apoptotic cell death, and plenty of swollen mitochondria (Fig. 12c). Treating with functionalized CEINs (Fe@C–(CH_2_)COOH) also induced internalization of iron nanomaterials in melanoma cells (Fig. 12d).

## Discussion

Preclinical studies evidence that malignant cells are more prone to internalize the MNPs than normal cells (Fisichella et al. 2010; Saltan et al. 2011). The reason is that malignant cells possess a higher endocytotic potential than that of normal cells due to their enhanced requirement for nutrients in virtue of their high metabolic activity and proliferation rate (Clement et al. 2006; Zhang et al. 2009). Recent data also suggest that once the MNPs are located onto the cellular surface in cancer cells, fast internalization probably begins (Dam et al. 2012; Lu et al. 2012; Raoof et al. 2012). This was found in agreement with our findings presenting a number of internalizations in melanoma cells incubated with CEINs. To data, our theoretical calculation of the SCD revealed that both Fe@C-COOH and Fe@C–CH_2_CH_2_–COOH have a large electrical surface charge as compared to the purified Fe@C samples in physiological pH, respectively (Table 1). The pH value of the PBS buffer (7.4) significantly exceeds the point of zero charge in the purified and surface functionalized CEINs. Importantly, the raw CEINs do not carry the surface charge, because their point of zero charge is almost the same as the pH of the PBS buffer. The SCD is therefore a really valuable parameter in nanoparticle cell studies, which express the mean charge value over the global surface area which is available to the surrounding environment (cell/medium). The surface area (in m^2^/g) values are typical for the non-porous sorbents and vary between 60 and 95 m^2^/g for all CEINs samples. The calculated SCD changes in the same order as the surface acidity (Table 2). To present these data more clearly, one can say that the individual CEIN particle from the sample Fe@C–CH_2_CH_2_–COOH statistically has three times larger number of surface acidic groups in comparison with the sample Fe@C–COOH, respectively. Hence, the surface acidic groups get the negatively charged when CEINs(COOH)*n* are in contact with cell membranes in the culture medium. To data, it is also possible to evaluate the average density of surface acidic groups in respective CEINs. This density can be expressed as an average number of surface groups per one hexagon in a graphitic lattice (the carbon coating in CEINs is built of curved graphene layers). The *D* values are shown in Table 2. The density of surface acidic groups increases with an increase of the total surface acidity and changes in the same order as the SCD. The density of surface acidic groups in sample Fe@C–(CH_2_)_2_COOH is the highest and in this case 3–4 ligands are attached to one hexagon in graphitic lattice.

In our conducted research, the MTT assay was used to measure the basic cytotoxicity of CEINs in melanoma and normal cells of the dermal origin. Interestingly, incubation of human normal dermal fibroblasts with non-functionalized raw iron nanoparticles produced significant loss in viability of about 55 and 75 % observed only at higher concentrations of 50 and 100 μg/ml, respectively. Below this concentration (0.0001–10 μg/ml), cellular metabolic activity did not change as much as in comparison with unaffected control (Fig. 6). In contrast, all iron nanoparticles affected the metabolic activity in concentration-dependent manner, but in different patterns, when they were added at the concentration range of 1–100 μg/ml to the cancer (melanoma) cells. In general, the cytotoxicity of CEINs has been increased in relation to increasing concentration, as shown in Figs. 4 and 5. Surface modified iron nanoparticles (Fe@C–COOH and Fe@C–CH_2_CH_2_–COOH) revealed weak cytotoxic effects to both human normal fibroblasts and human melanoma cells, as well, and they remained around 80–100 % viable. These samples decreased the viability of the murine cancer cells to about 40–60 % depending on the increased nanoparticle concentration in the medium.

In our cytotoxicity studies, carbon-encapsulated iron nanoparticles with surface acidic groups are found to be more hydrophilic forms than that of the non-functionalized CEINs. In contrast to raw and purified CEINs without surface functional groups, they show significantly higher cell-survival rates in all cells exposed at doses of 50 and 100 μg/ml but their toxicity plausibly depends highly on the magnitude of the surface charge. Recent data evidenced that hydrophilic surfaces of nanoparticles, such as dextran, polyethylene glycol, polyethylene oxide, poloxamers, polysorbates, and polyoxamines provide a dynamic “cloud” of hydrophilic and neutral chains at the nanoparticle surface (Chiappetta et al. 2011; Gillich et al. 2011; Karmali et al. 2012). In other studies the significant role of surface charge (both positive and negative) and nanomaterial type on the uptake and biocompatibility of nanoparticles was discussed (Chen et al. 2011; Sohaebuddin et al. 2010). It should be noted that the MNP uptake by cells due to the vesicular transport-based cell endocytosis could be considered as a binding process followed by an internalization process (Zhang et al. 2008). To explain this phenomenon, Zeta potential measurements have been utilized in studying the interaction between normal breast epithelium cells (MCF10A) and cancer breast epithelium (MCF7) after incubation with iron oxide nanoparticles for different regiments of treatments (Zhang et al. 2008). The changes in Zeta potential values were found to be relative to cell surface charge, nanoparticle surface charge, and the interaction between cells and iron oxide nanoparticles (Zhang et al. 2008). Although, we did not measure directly the Zeta potential value of the respective CEIN sample(s) as well as normal and cancer (melanoma) cells studied to data, a battery of alternative cytotoxicity tests including LDH leakage assays following Annexin V-FITC/PI and Calcein AM/PI labeling were applied for all the four types of carbon-encapsulated iron nanomaterials to study CEIN-related membrane cytotoxicity effects due to differences in SCD. Interestingly, in the present study, “subtle” differences in cytotoxicity effects between both non-modified and surface modified CEINs possessing surface acidic groups were noted in normal dermal fibroblasts and melanoma cancer cells. These results clearly support Zhang et al. (2008) observations that Zeta potential and SCD (present data) parameters could really affect internalization processes and therefore might play a role in cytotoxicity effects of MNPs in cells.

The LDH leakage assay relies on the membrane permeability of the cytosolic enzyme (lactic dehydrogenase) and represents a parameter for the cell integral stability. In the plasma membrane of the damaged or dead cells, the released LDH transfers lactate to pyruvate with co-reaction of NAD^+^ to NADH transition, and then the oxidation reaction of NADH to NAD^+^ due to diaphorase transfers the H/H^+^ from NADH/H^+^ to the tetrazolium salt, which is reduced to a formazan dye. In the LDH leakage assay, the amount of color formed is proportional to the number of lysed cells. Interestingly, viable cells produce negligible formazan dye signal with LDH leakage assay. The LDH leakage assay measures the signal of membrane-damaged cells, not the live normal ones. Therefore, even though the cell number is increasing as incubation time is 24 h, the LDH leakage assay cannot count the viability of live ones, but only detect the accumulated LDH derived from continuous leakage of damaged cells which number is proportional to the time. From the results of the LDH leakage test, we observed that all types of carbon-encapsulated iron nanoparticles induced different changes in normal and cancer cells, thereby suggesting that both human and murine melanoma cancer cells as well as human normal dermal fibroblasts probably had responded in different patterns to internalize the nanoparticles via cell membranes. Based upon the results of externalization of PS, which is localized commonly on the outer cellular membrane in cells, Annexin-V was also applied to determine the apoptotic rate of the tested cells in response to the treatment of CEINs. Analysis of Annexin V-FITC/PI stained cells by flow cytometry allows quantitation of the fraction of cells that are Annexin V-negative and propidium iodide-negative (double negative), Annexin V-positive and propidium iodide-negative (single positive), or Annexin V-positive and propidium iodide-positive (double positive). In the present study, human melanoma cells were found to be more sensitive to prone early apoptosis events due to CEINs as compared to that of murine melanoma cells as well as normal HDFs. In flow cytometry results, the population of Annexin V-positive live cells in the human melanoma cancer cell line increased, and most (29.3 %) of the cells became Annexin V-positive at 24 h at a concentration of 1 μg/ml for the raw CEIN sample (Fe@C/Fe). Interestingly, our results with Calcein AM/PI double staining also evidenced that especially murine melanoma cells respond in late apoptosis/necrosis patterns when dosed with CEINs. These both observations were found in agreement with flow cytometry studies (Fig. 11b) and other recent reports in which MNPs such as iron oxides and carbon-encapsulated iron nanoforms promoted both early and late apoptotic events in different cancer cell lines (Kai et al. 2011; Saltan et al. 2011; Xia et al. 2011). Realizing the future clinical potential of these novel magnetic nanoplatforms as new contrast drug candidates in mMRI, a more through approach has to be done to better understand their cytotoxic insults at the molecular level in details.

## Conclusion

The significance of this work is to elucidate subtle differences in cytotoxic effects between CEINs used as raw and purified materials, of which the carbon surface was functionalized with surface acidic groups. Results clearly demonstrate that CEINs plausibly interact with some mitochondrial and cell membranes as examined by MTT and LDH leakage assays and TEM analysis as well. Our flow cytometry studies with double staining (Annexin-V-FITC/PI) of normal and melanoma cells also evidenced that early apoptosis events are trigged when the raw and purified CEIN materials are used in both human cancer and normal cells. Results show that CEIN-cell interactions plausibly depend on the surface aspects of the magnetic nanomaterial, which may be described according to their surface chemistry, hydrophilic/hydrophobic characteristics, or SCD. The final conclusions on particular CEIN material-type pattern toxicities should be viewed very carefully due to the complexity of the mechanism(s) determining the potent interactions at the CEIN-melanoma cell interfaces. Therefore, further detail studies on carbon-encapsulated iron nanoparticles containing modified surface with specific bio-ligands such as monoclonal antibodies or peptides targeting specific molecular receptors in cells are needed to determine the cancer cell behavior on contact with CEINs. More through approaches have to be also done to study CEINs in preclinical animal cancer models.

## Electronic supplementary material

Below is the link to the electronic supplementary material.

Supplementary material 1 (DOCX 31 kb)
