# Supplementary material for: Cytotoxicity evaluation of carbon-encapsulated iron nanoparticles in melanoma cells and dermal fibroblasts
Source: J Nanopart Res. 2013 Jul 24;15(8):1835. doi: 10.1007/s11051-013-1835-7 (PMC3751228; doi:10.1007/s11051-013-1835-7)
Supplement: Supplementary file 1 — Supplementary material 1 (DOCX 31 kb) [file 11051_2013_1835_MOESM1_ESM.docx]

**Fig. S1.** Mass titration curves of raw (Fe@C/Fe), purified (Fe@C) and surface functionalized (Fe@C-COOH, Fe@C-(CH_2_)COOH) carbon-encapsulated iron nanoparticles.
